# Role of L-alanine for redox self-sufficient amination of alcohols

**DOI:** 10.1186/s12934-014-0189-x

**Published:** 2015-01-23

**Authors:** Stephanie Klatte, Volker F Wendisch

**Affiliations:** Chair of Genetics of Prokaryotes, Faculty of Biology & CeBiTec, Bielefeld University, Universitaetsstr. 25, 33615 Bielefeld, Germany

**Keywords:** Redox self-sufficient amination, Whole cell biotransformation, *Escherichia coli*, Transaminase, *Chromobacterium violaceum*, Energy maintenance, Acetate formation, Pyruvate oxidase, Phosphate acetyltransferase, Acetate kinase

## Abstract

**Background:**

In white biotechnology biocatalysis represents a key technology for chemical functionalization of non-natural compounds. The plasmid-born overproduction of an alcohol dehydrogenase, an L-alanine-dependent transaminase and an alanine dehydrogenase allows for redox self-sufficient amination of alcohols in whole cell biotransformation. Here, conditions to optimize the whole cell biocatalyst presented in (Bioorg Med Chem 22:5578–5585, 2014), and the role of L-alanine for efficient amine functionalization of 1,10-decanediol to 1,10-diaminodecane were analyzed.

**Results:**

The enzymes of the cascade for amine functionalization of alcohols were characterized *in vitro* to find optimal conditions for an efficient process. Transaminase from *Chromobacterium violaceum*, Ta_Cv_, showed three-fold higher catalytic efficiency than transaminase from *Vibrio fluvialis*, Ta_Vf_, and improved production at 37°C. At 42°C, Ta_Cv_ was more active, which matched thermostable alcohol dehydrogenase and alanine dehydrogenase and improved the 1,10-diaminodecane production rate four-fold. To study the role of L-alanine in the whole cell biotransformation, the L-alanine concentration was varied and 1,10.diaminodecane formation tested with constant 10 mM 1,10- decanediol and 100 mM NH_4_Cl. Only 5.6% diamine product were observed without added L-alanine. L-alanine concentrations equimolar to that of the alcohol enabled for 94% product formation but higher L-alanine concentrations allowed for 100% product formation. L-alanine was consumed by the *E. coli* biocatalyst, presumably due to pyruvate catabolism since up to 16 mM acetate accumulated. Biotransformation employing *E. coli* strain YYC202/pTrc99a-*ald*-*adh*-*ta*_Cv_, which is unable to catabolize pyruvate, resulted in conversion with a selectivity of 42 mol-%. Biotransformation with *E. coli* strains only lacking pyruvate oxidase PoxB showed similar reduced amination of 1,10-decanediol indicating that oxidative decarboxylation of pyruvate to acetate by PoxB is primarily responsible for pyruvate catabolism during redox self-sufficient amination of alcohols using this whole cell biocatalyst.

**Conclusion:**

The replacement of the transaminase Ta_Vf_ by Ta_Cv_, which showed higher activity at 42°C, in the artificial operon *ald-adh*-*ta* improved amination of alcohols in whole cell biotransformation. The addition of L-alanine, which was consumed by *E. coli* via pyruvate catabolism, was required for 100% product formation possibly by providing maintenance energy. Metabolic engineering revealed that pyruvate catabolism occurred primarily via oxidative decarboxylation to acetate by PoxB under the chosen biotranformation conditions.

**Electronic supplementary material:**

The online version of this article (doi:10.1186/s12934-014-0189-x) contains supplementary material, which is available to authorized users.

## Background

White biotechnology is the key technology for alternative and sustainable production of e.g. fine chemicals. Its application in biocatalysis is considered a branch of Green Chemistry which can replace or complement routes of chemical modification and functionalization. Enzymes catalyze reactions under mild conditions contrarily to chemical catalysts which often demands high pressure and temperature as well as toxic solvents. Among others, amine functionalization of chemical compounds is an important approach in biocatalysis to produce (poly)amines which are components of for example synthetics and coatings. This can be performed by amino acid dehydrogenases catalyzing NADH-dependent reductive amination of oxo-acids with ammonium or by transaminases transferring an amino group from a donor amine to a carbonyl compound. The cofactor pyridoxal-phosphate is covalently bound to the catalytic center of ω-transaminases to transfer the amino group to the acceptor molecule [1,2].

**Figure 1:**
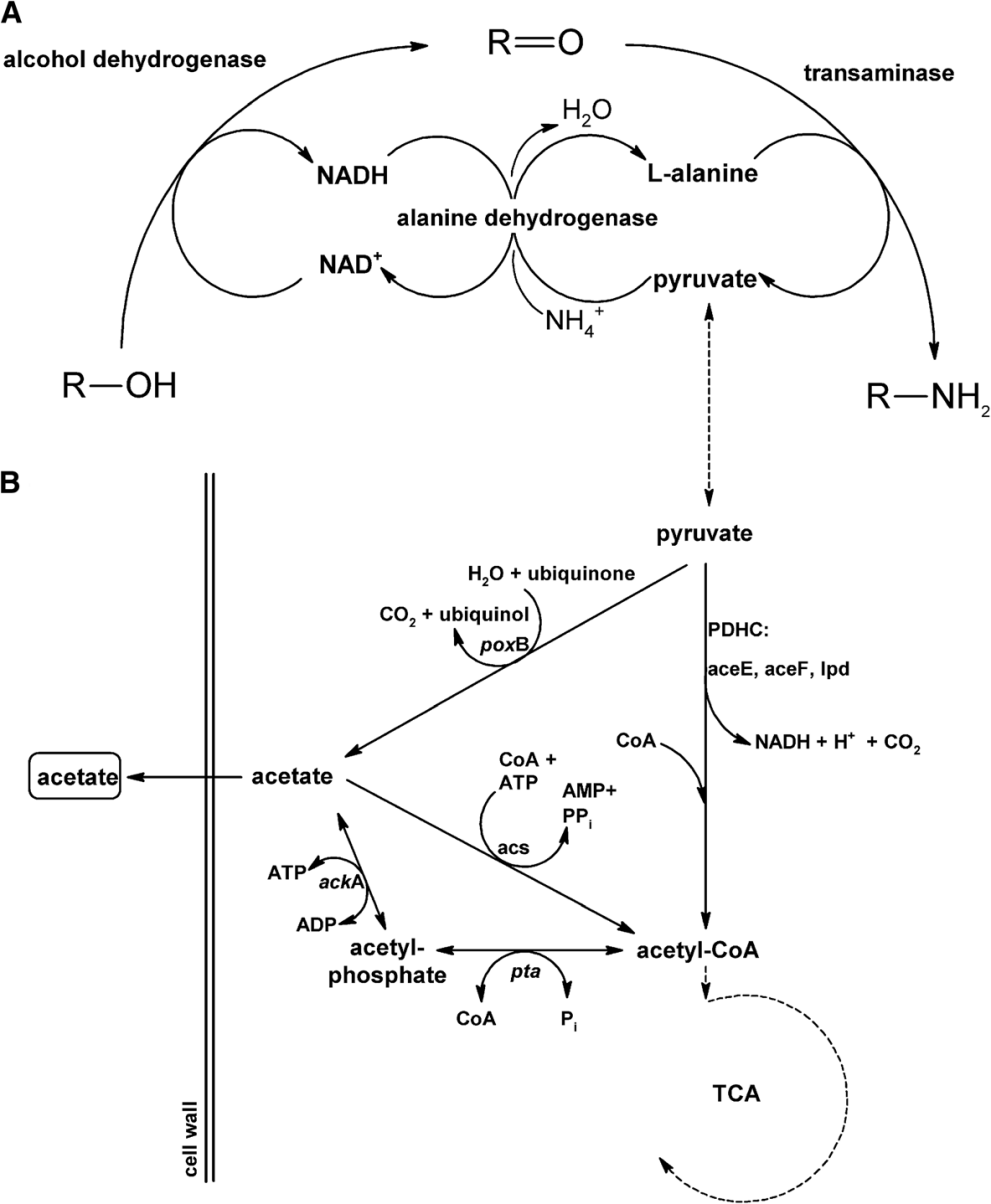
**Redox self-sufficient amination by coupling an alcohol dehydrogenase, L-alanine-dependent transaminase and L-alanine dehydrogenase (A); Catabolic reactions of** ***Escherichia coli*** **converting pyruvate (B).** For the reactions depicted in **(B)** gene names of pyruvate oxidase (*poxB*), the pyruvate dehydrogenase complex (*aceEFlpd*), acetyl-CoA synthetase (*acs*), phosphotransacetylase (*pta*) and acetate kinase (*ackA*) are given.

Generally, in biocatalysis the use of a transaminase instead of an amino-acid dehydrogenase offers the access to a wider range of substrates since amino-acid dehydrogenases accept a restricted spectrum of α-keto acids. Coupling of both types of enzymes enables amine functionalization of a wider range of carbonyl compounds from ammonium in a redox dependent manner. Therefore, redox cofactor recycling is important to reduce the process costs. Cofactor recycling by cascading with, for example, glucose dehydrogenase or formate dehydrogenase or for whole cell biocatalysts with cellular glucose catabolism have been developed [3,4]. A three enzyme cascade for recycling redox cofactor and amino group donor has been demonstrated in cell free biocatalysis using an alanine dehydrogenase coupled with a L-alanine-dependent transaminase and an alcohol dehydrogenase [5]. The initial alcohol oxidation by an alcohol dehydrogenase yields NADH and the aldehyde which is converted to the amine by L-alanine-dependent transamination (Figure 1A). Alanine dehydrogenase recycles L-alanine using ammonium and NADH releasing NAD^+^ as redox cofactor for the alcohol dehydrogenase. Recently, this concept of redox self-sufficient amination of alcohols (Figure 1A) was realized in a whole cell process with *Escherichia coli* by plasmid-born overproduction of the alcohol dehydrogenase of *Bacillus stearothermophilus*, the transaminase of *Vibrio fluvialis* and the alanine dehydrogenase of *Bacillus subtilis* [6]. Advantageously, the whole cell process only required alcohol, L-alanine and ammonium but neither NAD nor PLP. In principle, L-alanine addition is not required in the three-enzyme-cascade and amination of alcohols to amines should be possible with an ammonium salt. However, whole cells require energy to maintain viability as well as for transport processes e.g. substrate uptake and product export as well as protein synthesis under starvation or other stress conditions. Since non-growing cells are typically used for whole cell biotransformation, the non-growth-associated maintenance coefficient reflects the cell’s requirement to catabolize an energy substrate such as glucose to keep the cell’s viability and functionality. Therefore, the role of L-alanine in the whole cell biotransformation of alcohols to amines was analysed and conditions for optimizing the whole cell biocatalyst towards improved amino functionalization of alcohols were tested.

## Results

### Construction of the whole cell biocatalyst W3110/pTrc99A-*ald*-*adh*-*ta*_Cv_ and its comparison to W3110/pTrc99A-*ald*-*adh*-*ta*_Vf_*in vitro* and *in vivo*

The whole cell biocatalyst W3110/pTrc99A-*ald*-*adh*-*ta*_Vf_ was previously shown to enable redox self-sufficient amination of a variety of alcohols [6] and involved thermo-sensitive transaminase from *Vibrio fluvialis*. Due to the thermostable alcohol dehydrogenase of *B. stearothermophilus* an increased reaction temperature for redox self-sufficient amination was considered to improve the production rate. Therefore, the gene for the transaminase of *V. fluvialis* was replaced by the gene for the transaminase of *C. violaceum* in the IPTG-inducible vector pTrc99A-*ald*-*adh*-*ta*_Vf_. The vector was used to transform *E. coli* W3110 to yield the whole cell biocatalyst W3110/pTrc99A-*ald*-*adh*-*ta*_Cv_. Enzyme activity assays of the newly constructed whole cell biocatalyst revealed that all three genes were functionally expressed. The crude extracts displayed enzyme activities of 9.8 ± 1.1 and 0.55 ± 0.03 U/mg for the alanine dehydrogenase and the alcohol dehydrogenase, respectively, which was similar to the activities in W3110/pTrc99a-*ald*-*adh*-*ta*_Vf_ [6]. However, with (S)-(−)α-methylbenzylamine as substrate, the specific activities of transaminase Ta_Cv_ of 0.62 ± 0.01 in W3110/pTrc99a-*ald*-*adh*-*ta*_Cv_ were two-fold higher than that of Ta_Vf_ in W3110/pTrc99a-*ald*-*adh*-*ta*_Vf_.

The catalytic efficiencies of both transaminases were estimated with L-alanine as donor substrate and hexanal as acceptor substrate. Therefore, crude extracts of W3110/pTrc99a-*ta*_Vf_ and W3110/pTrc99a-*ta*_Cv_ were assayed in a 50 mM potassium-phosphate buffer pH 7.4 with constant hexanal and varying L-alanine concentrations at 37°C. The L-alanine-dependent transamination of hexanal to hexylamine could be detected for both transaminases and K_m_- and V_max_-values for Ta_Vf_ were 20 mM L-alanine and 0.3 U/mg, respectively, and 35 mM L-alanine and 2 U/mg, respectively, for Ta_Cv_ (Table 1). The catalytic efficiency reflected by the V_max_/K_m_-value was 3-fold higher for Ta_Cv_ than for Ta_Vf_ (0.06 compared to 0.02 U mg^−1^ mM^−1^).

**Table 1:** ***In vitro*** **estimation of the catalytic efficiencies of transaminases Ta**_**Vf**_ **and Ta**_**Cv**_ **with L-alanine and hexanal as substrates**

**Origin of the transaminase**	**K**_**m**_ **for L-alanine [mM]**	**V**_**max**_ **[U/mg]**	**V**_**max**_ **/K**_**m**_
*V. fluvialis*	20.00 ± 1.10	0.30 ± 0.01	0.02
*C. violaceum*	35.00 ± 2.20	2.00 ± 0.07	0.06

To compare the whole cell biocatalysts W3110/pTrc99A-*ald*-*adh*-*ta*_Cv_ and W3110/pTrc99A-*ald*-*adh*-*ta*_Vf_ for the redox-self-sufficient amination of alcohols conversion of the diol 1,10-decanediol was tested. The addition of 1,10-decanediol, 100 mM L-alanine and 100 mM NH_4_Cl resulted in 100% product formation employing the newly derived catalyst W3110/pTrc99a-*ald*-*adh*-*ta*_Cv_ (Table 2) as well as employing the previously designed W3110/pTrc99a-*ald*-*adh*-*ta*_Vf_ [6]. When the L-alanine concentration was reduced to 50 mM or 20 mM, production of 1,10-diaminodecane using W3110/pTrc99a-*ald*-*adh*-*ta*_Cv_ and W3110/pTrc99a-*ald*-*adh*-*ta*_Vf_ was reduced to 84% and 86%, respectively, and 73% and 50%, respectively (Table 2). HPLC analysis revealed that both strains consumed between 16 and 30 mM L-alanine (Table 2). Taken together, the newly constructed biocatalyst W3110/pTrc99a-*ald*-*adh*-*ta*_Cv_ showed higher activity and catalytic efficiency with respect to the transaminase and higher selectivity at 37°C and with low L-alanine concentrations.

**Table 2:** **Comparison of the redox self-sufficent amination of 1,10-decanediol by W3110/pTrc99a-** ***ald-adh-ta***_**Vf**_ **and W3110/pTrc99a-** ***ald-adh-ta***_**Cv**_ **with varying L-alanine concentrations**

**Strain**	**Biotransformation conditions**	**Reaction temperature [°C]**	**Max. conversion [%]**	**Alanine consumption [mM]**
**W3110/pTrc99a-** ***ald-adh-ta***_**Cv**_	100 mM alanine, 100 mM NH_4_Cl	37	100_12h_	30
	50 mM alanine, 100 mM NH_4_Cl		84_12h_	30
	20 mM alanine, 100 mM NH_4_Cl		86_8h_	16
**W3110/pTrc99a-** ***ald-adh-ta***_**Vf**_	100 mM alanine, 100 mM NH_4_Cl	37	100_24h_	30
	50 mM alanine, 100 mM NH_4_Cl		73_12h_	26
	20 mM alanine, 100 mM NH_4_Cl		50_12h_	18

### Influence of the reaction temperature on amination of 1,10-decanediol to 1,10-diaminodecane by W3110/pTrc99a-*ald*-*adh*-*ta*_Vf_ and W3110/pTrc99a-*ald*-*adh*-*ta*_Cv_

**Figure 2:**
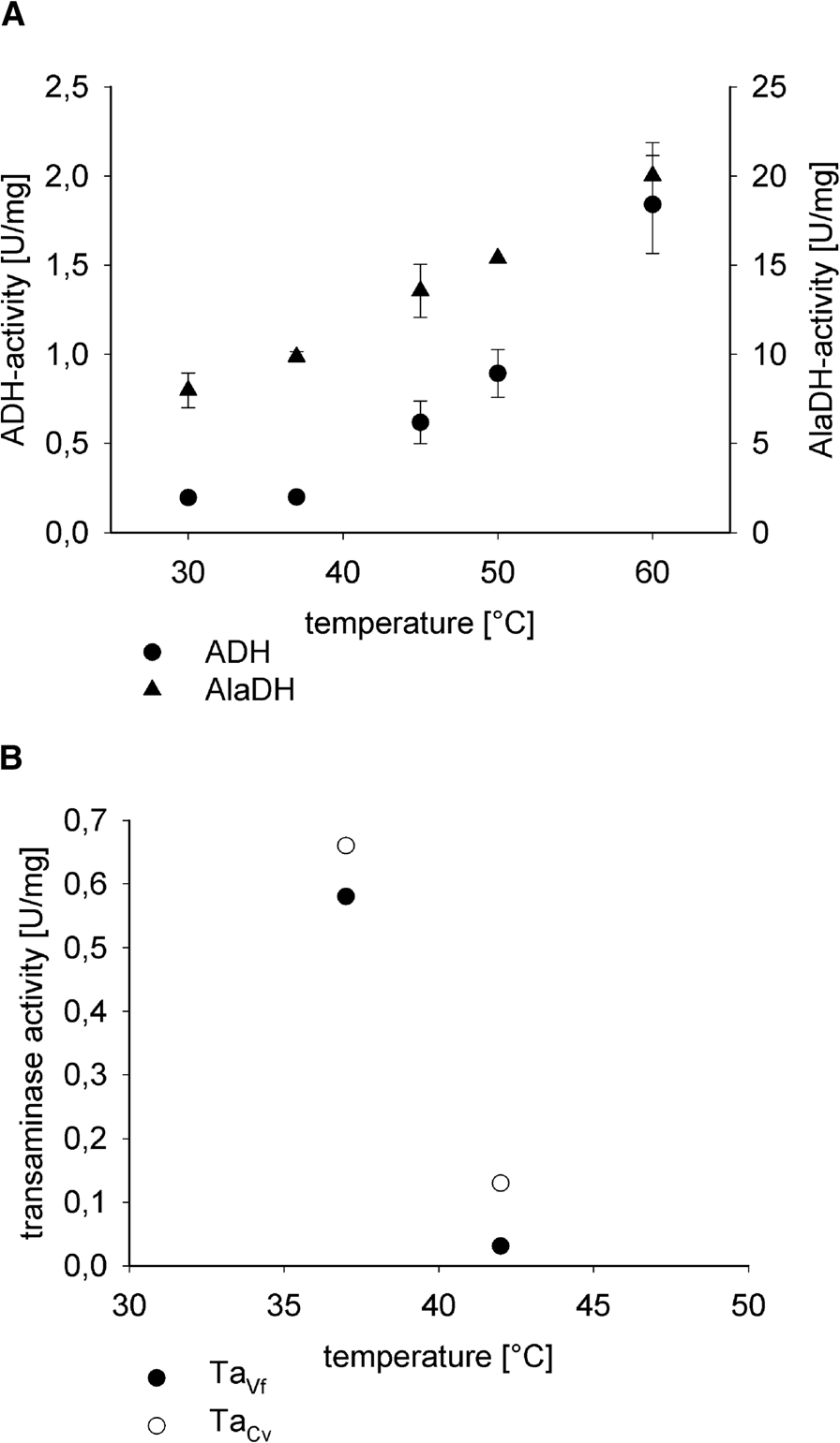
**Enzyme activity of the alanine dehydrogenase, alcohol dehydrogenase (A) and the transaminases of** ***Vibrio fluvialis*** **and** ***Chromobacterium violaceum*** **(B) measured in the crude extract of W3110/pTrc99a-** ***ald*** **, W3110/pTrc99a-** ***adh*** **, W3110/pTrc99a-** ***ta***_**Vf**_ **and W3110/pTrc99a-** ***ta***_**Cv**_ **, respectively, at different reaction temperatures.**

The three-enzyme-cascade contains thermostable alcohol dehydrogenase from the thermophilic *B. stearothermophilus*, but little is known about the activity of the alanine dehydrogenase from *B. subtilis* and the two transaminases of *V. fluvialis* and *C. violaceum* at higher temperatures. The activities of all enzymes were analyzed at varying temperatures in crude extracts of W3110/pTrc99a-*adh*, W3110/pTrc99A-*ald*, W3110/pTrc99a-*ta*_Vf_ as well as W3110/pTrc99a-*ta*_Cv_. For the alanine dehydrogenase of *B. subtilis* and the alcohol dehydrogenase of *B. stearothermophilus*, enzyme activities increased with temperature ranging from 30°C to 60°C and 20 U/mg and 1.8 U/mg, respectively, with maxima at about 60°C (Figure 2). The activity of the transaminase of *V. fluvialis* (Ta_Vf_) in the crude extract of W3110/pTrc99a-*ta*_Vf_ dropped from 0.5 U/mg to 0.03 U/mg when the reaction temperature increased from 37°C to 42°C. The activity of transaminase of *C. violaceum* (Ta_Cv_) was also lower at 42°C (0.13 U/mg) than at 37°C (0.66 U/mg), but its activity at 42°C was four-fold higher than that of Ta_Vf_ at 42°C (Figure 2).

**Figure 3:**
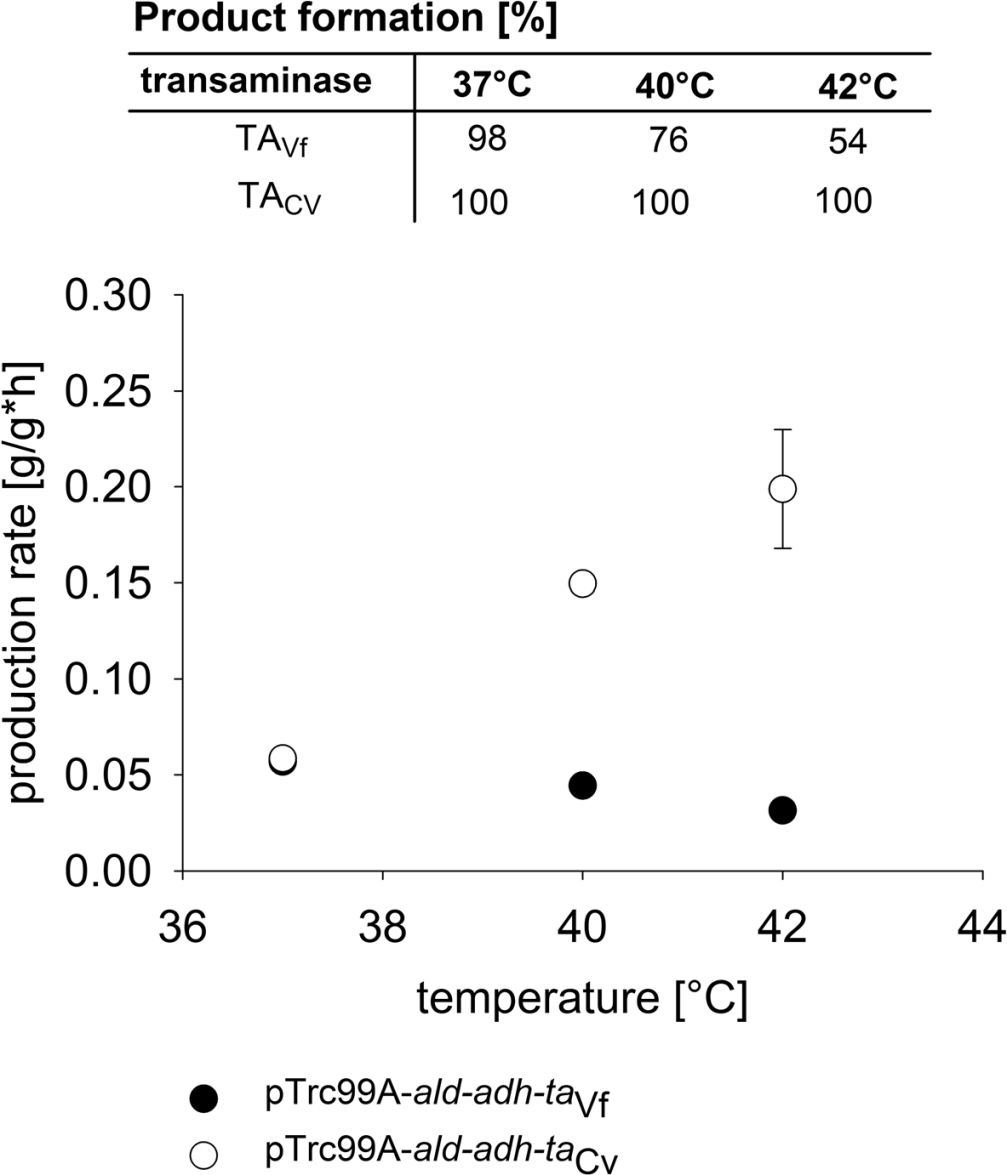
**Comparison of the production rate and product formation for redox self-sufficient amination of 1,10-decanediol at 37** **°C** **, 40** **°C** **and 42** **°C** **using whole cell biocatalysts W3110/pTrc99a-** ***ald*** **-** ***adh*** **-** ***ta***_**Vf**_ **and W3110/pTrc99a-** ***ald*** **-** ***adh*** **-** ***ta***_**Cv**_ **.** The cells were taken for whole cell biotransformation after 15 hours of gene expression in LB + 20 mM Mops at 37°C. In a resting system consisting of 50 mM Hepes pH7, 10 mM 1,10-decanediol, 100 mM L-alanine and 100 mM NH_4_Cl an optical density at 600 nm of 10 was adjusted.

To assay the influence of the reaction temperature on the amination cascade, whole cell biotransformations with W3110/pTrc99a-*ald*-*adh*-*ta*_Vf_ and W3110/pTrc99a-*ald*-*adh*-*ta*_Cv_ were performed at 37°C, 40°C and 42°C being suitable temperatures for the host *E. coli*. 1,10-decanediol was chosen as a substrate since it was among the best substrates of this cascade [5,7] and, moreover, the use of this dialcohol allows for monitoring 1-amino-10-decanol, an intermediate of diamine formation. The diamine production of both strains was compared in a resting buffer system using 1,10-decanediol as substrate, 100 mM L-alanine and 100 mM NH_4_Cl. *In vivo* amination of 1,10-decanediol performed by the two whole cell biocatalysts reached 100 mol-% selectivity after 24 hours at 37°C and production rates of about 0.06 g/g*h (0.35 mmole/g*h) were calculated (Figure 3). Furthermore, the increase of the reaction temperature from 37°C to 40°C and 42°C improved the production rates to 0.15 g/g*h (0.87 mmole/g*h) and 0.2 g/g*h (1.16 mmole/g*h), respectively, when the newly constructed W3110/pTrc99a-*ald*-*adh*-*ta*_Cv_ was employed. Contrarily, with W3110/pTrc99a-*ald*-*adh*-*ta*_Vf_ substrate conversion to 1,10-diaminodecane (74% and 56%, respectively) and production rates (0.04 g/g*h and 0.03 g/g*h, respectively) were lower at 40°C and 42°C, respectively, as compared to 37°C (Figure 3). Thus, under the chosen conditions W3110/pTrc99a-*ald*-*adh*-*ta*_Cv_ was superior to W3110/pTrc99a-*ald*-*adh*-*ta*_Vf_ at an elevated temperature.

### The role of L-alanine for the redox self-sufficient amination of alcohols in a whole cell process

L-alanine was a component of the hitherto described redox self-sufficient amination of alcohols with cascaded enzymes or as whole cell biotransformation [5,6] as well as in the experiments described above to serve as amino group donor in L-alanine-dependent transamination. However, conceptually redox self-sufficient amination of alcohols does not require the addition of L-alanine, but may proceed from ammonium only (Figure 1A). Thus, in order to find out if external addition of L-alanine is necessary to drive amination of 1,10-decanediol via this cascade, varying L-alanine concentrations were assayed using biocatalyst W3110/pTrc99a-*ald*-*adh*-*ta*_Cv_ at 42°C with a constant concentration of 100 mM NH_4_Cl. At reduced L-alanine concentrations of 50 mM and 20 mM complete conversion of 1,10-decanediol to 1,10-diaminodecane was observed after 6 hours. With 10 mM and 5 mM L-alanine selectivity was almost complete (94% and 93%, respectively), whereas only 5.6% conversion of 1,10-decanediol to 1,10-diaminodecane was observed without adding L-alanine (Table 3). Thus, L-alanine has to be present for efficient amination of alcohols using biocatalyst W3110/pTrc99a-*ald*-*adh*-*ta*_Cv_.

**Table 3:** **Whole cell biotransformation with W3110/pTrc99a-** ***ald*** **-** ***adh*** **-** ***ta***_**Cv**_ **at 42°C with 100 mM NH**_**4**_ **Cl and various L-alanine concentrations**

**Biotransformation conditions**		**Conversion [%]**	**L-alanine consumption**	**Acetate production**
**1,10-decanediol**	**L-alanine**		**[mM]**	**[mM]**
10 mM	50 mM	100_6h_	23	16
10 mM	20 mM	100_6h_	13	3
10 mM	10 mM	94_12h_	10	0
10 mM	5 mM	93_12h_	5	0
10 mM	0 mM	5,6_8h_	0	0
0 mM	20 mM	0_12h_	20	2
10 mM	0 mM +20 mM pyruvate	70_4h_	7	7

L-alanine added was utilized completely (at 5 and 10 mM) or partially (at 20 and 50 mM) during the whole cell biotransformation approach. Under certain conditions, acetate accumulated as by-product. When L-alanine was present at the same or lower concentrations as the substrate 1,10-decanediol, acetate accumulation was not observed, however, at higher L-alanine excess increasing acetate concentrations could be observed (Table 3). L-alanine (20 mM) was catabolized entirely in the absence of the substrate 1,10-decanediol. Pyruvate only partially replaced L-alanine since only 70% product formation were detected with 20 mM pyruvate. Taken together, these results indicate L-alanine consumption and acetate formation by the host’s central carbon metabolism.

**Figure 4:**
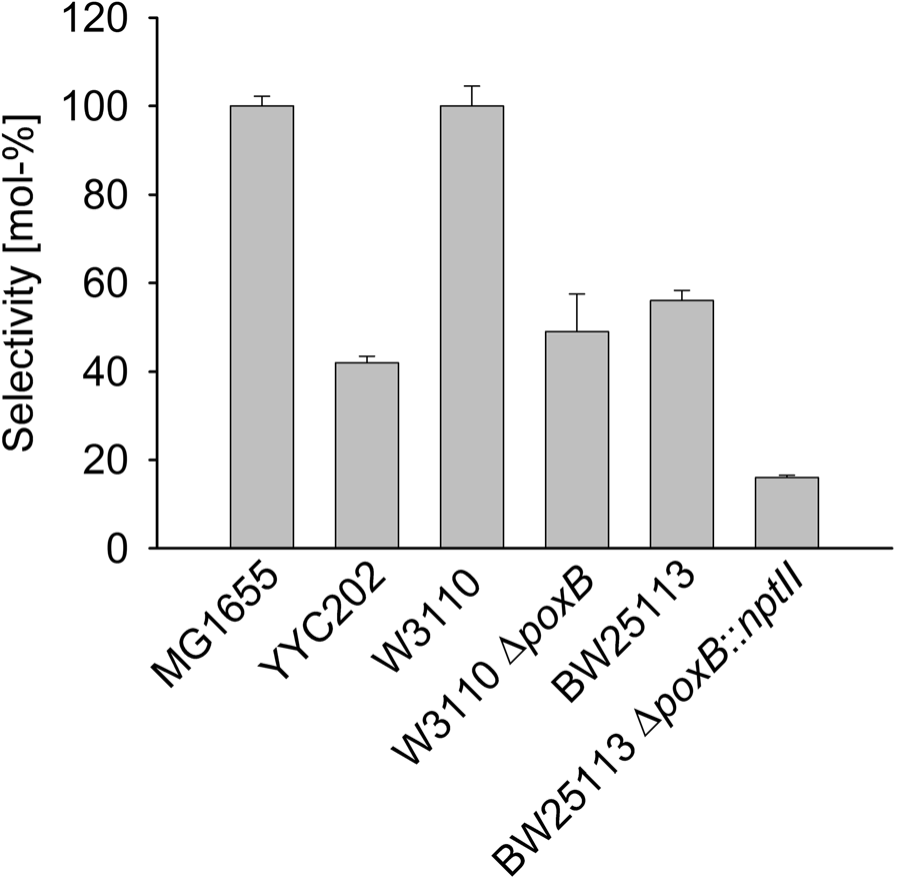
**Role of pyruvate oxidase PoxB for conversion of 1,10-decanediol to 1,10-diaminodecane in redox self-sufficient whole cell amination.** Amination of 1,10-decanediol was performed in a resting buffer system with 20 mM L-alanine, 100 mM NH_4_Cl and 10 mM 1,10-decanediol at 42°C. Strain YYC202 lacks *poxB* (and others) compared to its isogenic parent MG1655, strain W3110Δ*poxB* lacks *poxB* compared to its isogenic parent W3110 and BW25113Δ*poxB* lacks *poxB* compared to its isogenic parent BW25113. All strains carried pTrc99a-*ald*-*adh*-*ta*_Cv_.

To test if L-alanine consumption is important for the redox self-sufficient amination of alcohols with this whole cell biocatalyst or a dispensable side reaction, the host’s pyruvate catabolism was blocked at various positions. *E. coli* YYC202 cannot catabolize pyruvate (Figure 1) since it lacks the pyruvate dehydrogenase complex (deletion of *aceEF*), phosphoenyl pyruvate synthetase (mutation of *pps*), pyruvate formate lyase (*pflB*) and pyruvate oxidase (*poxB*) [7]. Heterologous overexpression of *ald*, *adh* and *ta*_Cv_ in *E. coli* YYC202 led to 42% conversion of 1,10-decanediol to 1,10-diaminodecane as compared to 100 mol-% selectivity obtained with the reference strain MG1655/pTrc99a-*ald*-*adh*-*ta*_Cv_ (Figure 4). Since there are multiple metabolic blocks in *E. coli* YYC202, additional strains were analysed to identify which of the reactions blocked in *E. coli* YYC202 is most important for pyruvate catabolism. It is generally believed that pyruvate dehydrogenase complex (PDHC) is important under aerobic conditions and pyruvate formate lyase (PFL) under anoxic conditions while PoxB might be important during the transition between exponential and stationary phase since under microaerobic conditions both PDHC and PFL function poorly [8]. PoxB also contributes to aerobic growth especially at low grow rates [9]. It is known that the pyruvate oxidase gene *poxB* is induced in the stationary growth phase and this induction is dependent on the alternative sigma factor RpoS [10]. Thus, PoxB might be relevant under the conditions of whole cell biotransformation that are characterized by slow growth and limited oxygen. Therefore, *poxB* was deleted in *E. coli* W3110 (s. Materials and method) and the resulting mutant was transformed with pTrc99a-*ald*-*adh*-*ta*_Cv_. The whole cell biocatalyst lacking only *poxB,* W3110Δ*poxB*/pTrc99a-*ald*-*adh*-*ta*_Cv_, showed about two-fold reduced amination of 1-10-decanediol to 1,10-diaminodecane (Figure 4) indicating that PoxB is the most important enzyme for pyruvate catabolism under the chosen biotransformation conditions. Moreover, since *E. coli* strain differences have been observed to affect biotransformation [11], which may be relevant even between the closely related strains W3110 and MG1655 used here [12], the effect of the lack of PoxB was also analysed in a different strain background, namely BW25113. Indeed, *E. coli* BW25113/pTrc99a-*ald*-*adh*-*ta*_Cv_ converted about three times more (56%) 1,10-decanediol to 1,10-diaminodecane than its *poxB* mutant (16%; Figure 4). Taken together, the addition of L-alanine and its catabolism via pyruvate and pyruvate oxidase PoxB is important for efficient amino functionalization of alcohols by the described whole cell biocatalysts.

## Discussion

Redox self-sufficient amination of alcohols by whole cell biotransformation benefitted from replacing the transaminase from *V. fluvialis* used previously [6] by transaminase Ta_Cv_ from *C. violaceum* [13] since it showed higher activity at 42°C. Moreover, it showed higher catalytic efficiency with L-alanine as substrate (Table 1). The low activities of transaminases at 42°C appeared to be limiting the efficiency of the whole cell biocatalyst since alcohol dehydrogenase of *B. stearothermophilus* and L-alanine dehydrogenase are rather thermostable (Figure 2) [14,15]. Indeed, Ta_Cv_, which showed higher activity at 42°C than Ta_Vf_, allowed for efficient conversion of 1,10-decanediol to 1,10-diaminodecane at 42°C. Shifting the biotransformation temperature from 37°C to 42°C led to about three-fold faster conversion employing W3110/pTrc99a-*ald*-*adh*-*ta*_Cv_ (Figure 3). Since at 37°C both W3110/pTrc99a-*ald*-*adh*-*ta*_Cv_ and W3110/pTrc99a-*ald*-*adh*-*ta*_Vf_ showed comparable production rates, the lower catalytic efficiency of Ta_Vf_ did not limit product formation under these conditions.

Addition of L-alanine was required for full and fast conversion of 1,10-decanediol to 1,10-diaminodecane using the whole cell biocatalyst for alcohol amination. The consumption of L-alanine over time suggested insufficient L-alanine recycling and loss of pyruvate via the cellular catabolism (Figure 1B; Table 3). This was less pronounced at higher 42°C possibly because under these conditions activities of both L-alanine dehydrogenase and alcohol dehydrogenase were increased allowing for more efficient redox cofactor recycling.

L-alanine served two functions in the biotransformation: As substrate in the transaminase reaction and to provide energy and reduction equivalents to the whole cell biocatalyst by catabolism of pyruvate, the co-product of L-alanine-dependent transamination (Figure 1). Under the non-growth conditions of whole cell biotransformation up to 20 mM of alanine were consumed (Table 3) with a rate of about 0.04 g/g*h, which is in the same order of magnitude as non-growth maintenance energy (0.055 to 0.07 g of glucose/g*h). Pyruvate addition only partially replaced L-alanine addition since product formation in the presence of 20 mM pyruvate was incomplete (70%; Table 3). In part, L-alanine was catabolized to acetate. If pyruvate is oxidatively decarboxylated to acetate by pyruvate oxidase PoxB, a reduction equivalent (ubiquinol) is formed which may be used (indirectly) for reductive amination by L-alanine dehydrogenase in the cascade. *E. coli* is known to produce acetate as overflow metabolite even under fully aerobic conditions, e.g. with excess glucose [16] when 10% - 30% of carbon flux is directed to acetate formation [17]. Acetate may be formed under aerobic growth conditions by the combined activities of pyruvate dehydrogenase complex PDHC, phosphotransacetylase Pta and acetate kinase AckA (Figure 1B). Besides the reduction equivalent NADH, this pathway yields ATP. A third pathway may be active as a mutant devoid of *poxB*, *pta* and *ackA* still produced acetate [18]. Fast catabolism of glucose to acetate followed by its reuse via acetyl-CoA synthetase (Figure 1B) may be advantageous in comparison to other microorganisms present in its natural habitat that slowly convert the limiting carbon source glucose [9]. In the absence of pyruvate oxidase PoxB conversion of 1,10-decanediol to 1,10-diaminodecane was reduced in about the same way as when PoxB and all other known enzymes for pyruvate degradation were missing, thus, indicating that PoxB is the major enzyme for pyruvate degradation under the chosen biotransformation conditions. In the biotransformation described here, the whole cell biocatalysts were harvested in the stationary phase when PoxB dominates.

## Conclusions

The newly derived whole cell biocatalyst W3110/pTrc99a-*ald*-*adh*-*ta*_Cv_ allowed for the improvement of redox self-sufficient amination of alcohols displayed by an increase in production rate. This was achieved by replacing the transaminase of *V. fluvialis* by the transaminase of *C. violaceum,* which showed higher activity at 42°C. The whole cell biocatalyst for redox self-sufficient amination of alcohols required L-alanine in concentrations equimolar to the dialcohol substrate for complete conversion to the diamine. L-alanine catabolism occurred primarily via pyruvate oxidase PoxB under the biotranformation conditions.

## Materials and method

### Bacterial strains, plasmids and oligonucleotides

The *E. coli* strains, plasmids and oligonucleotides used in this study are listed in Table 4.

**Table 4:** **Strains, plasmids and oligonucleotides used in this study**

**Strains**	**Relevant characteristics**	**Reference**
*E. coli* DH5α	F^−^ *thi*-1 *endA*1 *hsdr*17(r^−^, m^−^) *supE*44 Δ*lacU*169	[19]
	(ɸ80*lacZ*ΔM15) *recA*1 *gyrA*96 *relA*1	
*E. coli* W3110	F^−^ λ^−^ INV(*rrn*D – *rrn*E)1	[19]
*E. coli* MG1655	F^−^ λ^−^ *ilvG*- *rfb*-50 *rph*-1	[19]
*E. coli* YYC202	Δ*ace*EF *pfl*1 *pox*B1 *pps*4 *rps*L *zbi*::Tn10	[8]
*E. coli* BW25113	*lacI* ^q^ *rrnB*_T14_ *lacZ*_WJ16_ *hsdR*514 *araBA*-*D*_AH33_ *rhaBAD*_LD78_	[20]
W3110/pTrc99A-*ald*-*adh*-*ta*_Vf_	*E. coli* W3110 harboring pTrc99A-*ald-adh-ta* with the transaminase of *Vibrio fluvialis*	[6]
W3110/pTrc99A-*ald*-*adh*-*ta*_Cv_	*E. coli* W3110 harboring pTrc99A*-ald-adh-ta* with the transaminase of *Chromobacterium violaceum*	This study
MG1655/pTrc99A-*ald*-*adh*-*ta*_Cv_	*E. coli* MG1655 harboring pTrc99A-*ald-adh-ta* with the transaminase of *Chromobacterium violaceum*	This study
YCC202/pTrc99A-*ald*-*adh*-*ta*_Cv_	*E. coli* YCC202 harboring pTrc99A-*ald-adh-ta* with the transaminase of *Chromobacterium violaceum*	This study
BW25113/pTrc99A-*ald*-*adh*-*ta*_Cv_	*E. coli* BW25113 harboring pTrc99A-*ald-adh-ta* with the transaminase of *Chromobacterium violaceum*	This study
JW0855-1/pTrc99A-*ald*-*adh*-*ta*_Cv_ (BW25113Δ*poxB*::kan)	F-, *Δ(araD-araB)567*, *ΔlacZ4787*(::rrnB-3), *λ* ^*−*^, *ΔpoxB772::kan*, *rph-1*, *Δ(rhaD-rhaB)568*, *hsdR514; harboring the plasmid* pTrc99A-*ald-adh-ta* with the transaminase of *Chromobacterium violaceum*	[20]/Transformation in this study
JW2293-1/pTrc99A-*ald*-*adh*-*ta*_Cv_ (BW25113Δ*ackA*::kan)	F-, *Δ(araD-araB)567*, *ΔlacZ4787*(::rrnB-3), *λ* ^*−*^, *ΔackA778::kan*, *rph-1*, *Δ(rhaD-rhaB)568*, *hsdR514; harboring the plasmid* pTrc99A-*ald-adh-ta* with the transaminase of *Chromobacterium violaceum*	[20]/Transformation in this study
JW2294-1/pTrc99A-*ald*-*adh*-*ta*_Cv_ (BW25113Δ*pta*::kan)	F-, *Δ(araD-araB)567*, *ΔlacZ4787*(::rrnB-3), *λ* ^*−*^, *Δpta779::kan*, *rph-1*, *Δ(rhaD-rhaB)568*, *hsdR514; harboring the plasmid* pTrc99A-*ald-adh-ta* with the transaminase of *Chromobacterium violaceum*	[20]/Transformation in this study
**Plasmids**	**Relevant characteristics**	**Reference**
pTrc99A-*ta*_*Vf*_	pTrc99A carrying *ta* of *Vibrio fluvialis*	This study
pTrc99A-*ta*_*Cv*_	pTrc99A carrying *ta* of *Chromobacterium fluvialis*	This study
pTrc99A-*ald-adh-ta*_Vf_	pTrc99A carrying *ald-adh-ta*_Vf_ synthetic operon	[6]
	*ald* from *B. subtilis* 168	
	*adh* from *B. stearothermophilus*	
	*ta* from *V. fluvialis*	
pTrc99A-*ald-adh-ta*_Vf__mut	pTrc99A-*ald-adh-ta*_Vf_ with *Bam*HI cut site upstream of *ta*_Vf_	This study
pTrc99A-*ald-adh-ta*_Cv_	pTrc99A carrying *ald-adh-ta*_Cv_ synthetic operon	This study
	*ald* from *B. subtilis* 168	
	*adh* from *B. stearothermophilus*	
	*ta* from *C. violaceum*	
**Oligonucleotides**	**Sequence 5′→ 3′**	**Use**
ta_Vf__RBS_for	CAGACCATGGAATTCGAGCAGGAAACAGACCATGAACAAACCGCAGAGCTG	pTrc99A-*ta*_*Vf*_
ta_Vf__rev	ATCCCCGGGTACCGAGTTACGCAACTTCCGCGAAAAC	pTrc99A-*ta*_*Vf*_
ta_Cv__KpnIRBS_for	CAA*GGTACC*CAGGAAACAGACCATGCAGAAACAGCGTACCACC	pTrc99A-*ta*_*Cv*_
ta_Cv__BamHI_rev	GTT*GGATCC*TTAGGCCAGACCACGTGCTTTC	pTrc99A-*ta*_*Cv*_
pTrc99a-ald-adh-ta_Vf__mut_for	GGAAGATAAATAA*GGATCC*CAGGAAACAGACCATGAAC	pTrc99A-*ald-adh-ta*_Cv_
pTrc99a-ald-adh-ta_Vf__mut_rev	CTG*GGATCC*TTATTTATCTTCCAGGGTCAGAACAACA	pTrc99A-*ald-adh-ta*_Cv_
pTrc99a-ald-adh-ta_Cv_ _for	CAA*GGATCC*CAGGAAACAGACCATGCAGAAACAGCGTACCACC	pTrc99A-*ald-adh-ta*_Cv_
pTrc99a-ald-adh-ta_Cv_ _rev	GTT*GGATCC*TTAGGCCAGACCACGTGCTT	pTrc99A-*ald-adh-ta*_Cv_

Competent cells and vector cloning was performed according to standard DNA work procedure [21]. In this study two different cloning strategies for *E. coli* expression vectors based on IPTG-inducible pTrc99a were used. Firstly, cut sites were used for inserting a gene into a vector. Therefore, PCR-derived gene product *ta* of *Chromobacterium violaceum* [GI: 34105712; codon-optimized] (ta_Cv__KpnIRBS_for; ta_Cv__BamHI_rev) amplified by KOD Hot Start Polymerase Kit (Novagen) was cut with *Kpn*I and *Bam*HI and used for ligation with also *Kpn*I and *Bam*HI treated pTrc99A to generate pTrc99a-*ta*_Cv_. To construct pTrc99a-*ald*-*adh*-*ta*_Cv_ the *Bam*HI cut site was inserted upstream of *ta*_Vf_ within the artificial operon *ald*-*adh*-*ta*_Vf_ of pTrc99a- *ald*-*adh*-*ta*_Vf_ by site directed mutagenesis using the oligonucleotides pTrc99a-ald-adh-ta_Vf__mut_for [22]. The newly derived vector pTrc99a-ald-adh-ta_Vf__mut was then cut by BamHI and ligated with *Bam*HI cut *ta* of *Chromobacterium violaceum* that was amplified by KOD Hot Start Polymerase Kit (Novagen, pTrc99a-ald-adh-ta_Cv_ _for, pTrc99a-ald-adh-ta_Cv__rev). Secondly, to construct pTrc99A-*ta*_Vf_ the gene *ta* of *Vibrio fluvialis* was amplified with the oligonucleotides ta_Vf__RBS_for and ta_Vf__rev and assembled with EcoICRI restricted pTrc99a using Gibson assembly method [23]. Then, *E. coli* DH5α was transformed with the ligation products. CaCl_2_-competent *E. coli* DH5α were heat-shocked for the uptake of ligation products. Newly derived vectors were proven by sequencing and *E. coli* W3110 was transformed with correct plasmids pTrc99a-*ta*_Vf_, pTrc99a-*ta*_Cv_ and pTrc99A-*ald-adh-ta*_Cv_ and *E. coli* MG1655 as well as *E. coli* YCC202 with pTrc99A-*ald-adh-ta*_Cv_.

### Cultivation conditions and media

Standard cultivation of *E. coli* was performed in Luria-Bertani medium (LB-medium: 10 g/L NaCl, 10 g/L tryptone, 5 g/L yeast extract) at 37°C and 200 rpm in baffled flasks or plated on LB-Agar as it is not declared otherwise. When strains harboring plasmid pTrc99A and its derivatives, 100 μg/mL ampicillin was supplemented to the medium. Strain YYC202 and its derivatives were supplemented with 25 μg/ml streptomycin and 10 μg/ml tetracycline, additionally.

### Preparation of cell free extract and enzyme assay

The *E. coli* derivatives were grown in LB + 100 μg/mL ampicillin until an optical density at 600 nm of 0.6 – 0.8, induced with 1 mM isopropyl-β-D-thiogalactopyranosid (IPTG) and harvested in the exponential phase at OD_600_ = 3.5. 10 mL of the cell culture was harvested and always kept on ice. The cells were once washed with buffer for the enzyme assay, resuspended in 1 mL of the same buffer and then lysed by sonication (Ultrasonic processor UP200S, Hielscher Ultrasound Technology, Teltow, Germany) for 2 minutes (cycle 0.5; amplitude 55%). Cell cebris was centrifuged at 10.000 x g at 4°C for 1 hour and clear cell extract was used for the measurement of the enzyme activity.

#### Measurement of alanine dehydrogenase activity

50 mM Na_2_CO_3_ pH 10 was used for cell washing. For measuring the activity of alanine dehydrogenase pyruvate was converted to L-alanine by NADH consumption spectrophotometrically at 340 nm. Therefore, 50 mM Na_2_CO_3_ pH 8.5, 50 mM NH_4_Cl, 10 mM pyruvate and 0.25 mM NADH where mixed in a cuvette, filled up to 1 mL ddH_2_O and upon the addition of crude extract the reductive amination was initiated and measured for 3 minutes. The assay was performed in triplicates and one enzyme unit was calculated to be the amount of enzyme that catalyzes the conversion of 1 μmol substrate in 1 min.

#### Measurement of alcohol dehydrogenase activity

25 mM Sodium phosphate buffer pH 8 was used for cell washing. For measuring the alcohol dehydrogenase activity 1,4-butanediol was oxidized to hydroxybutyraldehyde and NADH formation followed spectrophotometrically at 340 nm. Therefore, 25 mM Na-P-buffer pH 8, 18 mM 1,4 butanediol and 10 mM NAD^+^ were mixed in a cuvette, filled up to 1 mL ddH_2_O and reaction was initiated upon the addition of crude extract (triplicates). The NADH formation was followed over 3 minutes and one enzyme unit was calculated to be the amount of enzyme that catalyzes the conversion of 1 μmol substrate in 1 min. To analyze substrate specificity to 1-hexanol, 1-octanol, 1,6-hexanediol, 1,8-hexanediol, cyclohexanol, benzylalcohol and 2-hexanol same conditions were used but different substrate concentration were added to estimate K_m_ and V_max_-values via Lineweaver-Burk Plot.

#### Measurement of transaminase activity

100 mM Potassium-phosphate buffer pH 7.4 was used for cell washing. Reaction conditions were: 100 mM K-P-buffer pH 7.4, 50 mM (S)-α-MBA and 10 mM pyruvate. The transamination was initiated upon the addition of crude extract and samples were taken continuously. The reaction was stopped with 75 μl 16% perchloracetic acid. The samples where neutralized by the addition of 40 μl buffer containing 20 mM Tris/HCl pH 8 and 23 mM K_2_CO_3_. L-alanine formation was measured via HPLC and one enzyme unit was calculated to be the amount of enzyme to catalyze the formation of 1 μmol product in 1 min.

The experimental procedure for the estimation of catalytic efficiency was equal but hexanal was used as substrate instead of (S)-α-MBA. The Reaction conditions were: 100 mM K-P-buffer pH 7.4, 10 mM hexanal and varying concentrations of L-alanine. Hexylamine formation was measured via HPLC and one enzyme unit was calculated to be the amount of enzyme to catalyze the formation of 1 μmol product in 1 min.

### Whole cell biotransformation with resting cells

*E. coli* W3110/pTrc99A and its derivatives W3110/pTrc99A-*ald-adh-ta* and W3110/pTrc99A-*ta-ald-adh* were inoculated to an initial OD_600_ = 0.1 in LB-medium plus 20 mM Mops and 100 μg/mL ampicillin and incubated at 37°C and 200 rpm. At an OD_600_ = 0.6-0.8 1 mM IPTG was added to the expression culture to induce the cells and cultivation was continued as described above. 15 hours cells were harvested for a final OD_600_ = 10 in 20 mL final volume, once washed with 50 mM Hepes buffer pH 7 and prepared for whole cell biotransformation in a resting buffer system with the mentioned buffer. NH_4_Cl and L-alanine were added to the system when necessary and concentrations are given in the text. The test reaction containers (100 mL Schottbottle) where incubated at 37°C or 42°C and 200 rpm and samples for HPLC-analytics were taken in intervals throughout the production.

### HPLC-analysis

Extracellular amines and 1-amino-10-decanol were analyzed by high-pressure liquid chromatography (HPLC, 1200 series, Agilent Technologies Deutschland GmbH, Böblingen, Germany). Samples were centrifuged at 10.000 × g for 5 minutes and the clear supernatant was taken for HPLC-measurement. For the detection samples were derivatized with *ortho*-phthaldialdehyde (OPA) automatically before entering the precolumn (LiChrospher 100 RP8 EC-5 μ, 40 × 4.6 mm, CS-Chromatographie Service GmbH, Langerwehe, Germany) and the main column (LiChrospher 100 RP8 EC-5 μ, 125 × 4.6 mm, Langerwehe, Germany) for separation. The used mobile phase was made of A: 0.25% (v/v) Na-acetate buffer pH 6 and B: Methanol; 0 min 30% B, 1 min 30% B, 8 min 70% B, 13 min 90% B, 16 min 70% B, 18 min 30% B. 1,7-diaminoheptane was used as internal standard.

The detection of amino acids were performed with a quicker HPLC-method but derivatization with OPA was used equally to amine detection. Here, through a precolumn (LiChrospher 100 RP 18–5 EC; 40 × 4 mm) and the main column (LiChrospher 100 RP18 EC-5 μ; 125 × 4.6 mm; CS-Chromatographie Service GmbH, Langerwehe, Germany) amino acids were separated and detected by a FLD-detector. As an internal standard L-asparagine was used and the gradient for improved separation was made of A: 100 mM Sodiumacetate pH 7.2 and B: Methanol; 0 min 25% B, 0.5 min 45% B, 4 min 65% B, 7 min 70% B, 7.2 min 80% B, 7.4 min 85% B, 8 min 20% B, 10.6 min 20% B.

Overflow metabolites were separated by the Organic Acid Resin column (800 × 8 mm) from CS-Chromatographie Service GmbH (Langerwehe, Germany) and detected with DAD-detector. An isocratic elution with 5 mM H_2_SO_4_ and a flow rate of 0.7 ml/min for the separation of the samples.
